# Supplementary figures and images for: Seasonal influenza a virus lineages exhibit divergent abilities to antagonize interferon induction and signaling
Source: PLoS Pathog. 2024 Dec 12;20(12):e1012727. doi: 10.1371/journal.ppat.1012727 (PMC11637315; doi:10.1371/journal.ppat.1012727)

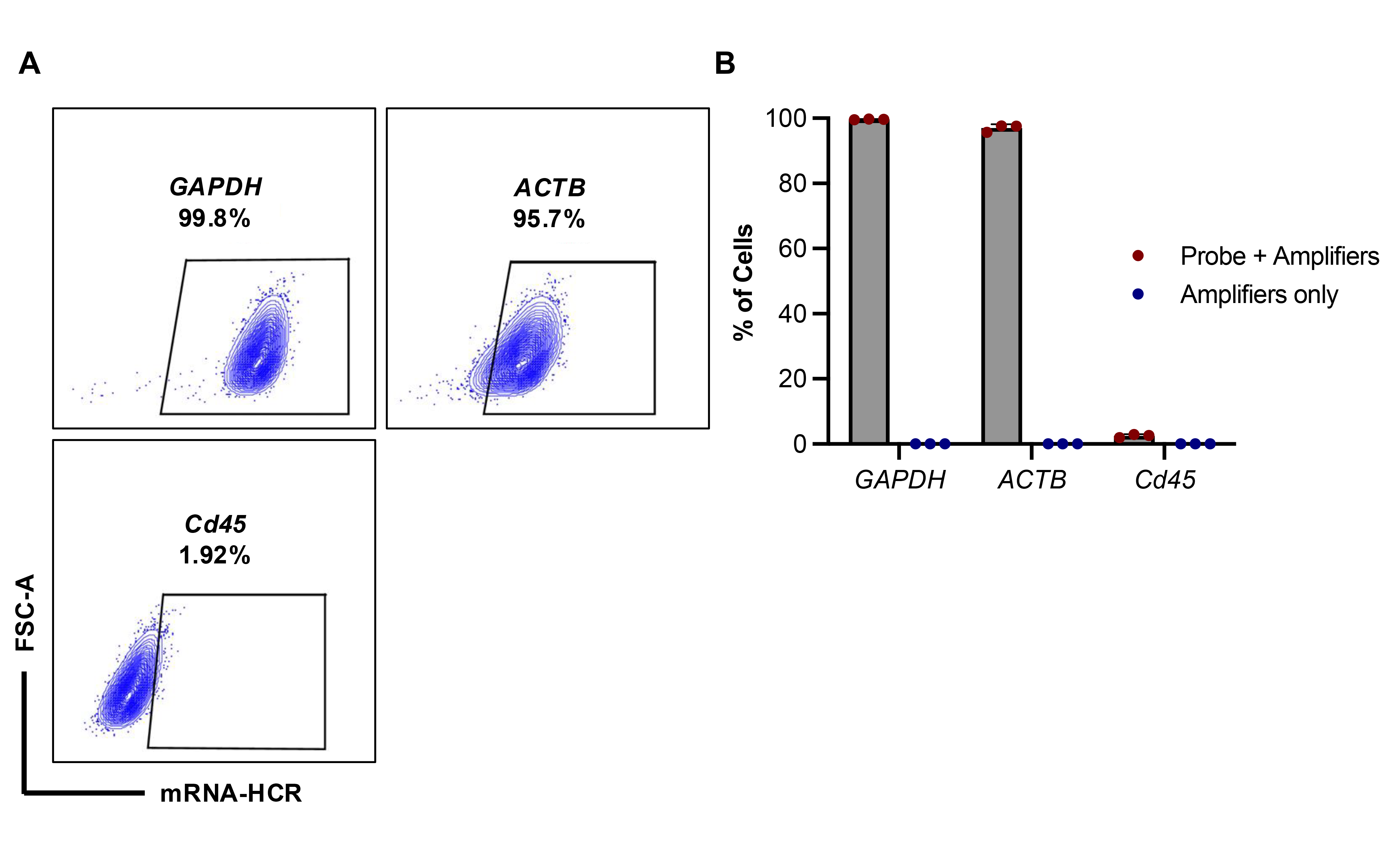

Supplement: S1 Fig — (A) Flow cytometry quantification of A549 cells stained for human GAPDH, ACTB, and mouse CD45 mRNA using HCR-flow. (B) Percentage of cells expressing housekeeping genes and mCD45 measured by flow cytometry in both cells stained using gene specific probes and matching amplifiers or only amplifiers. Data are shown as mean with SD; N = 3 cell culture wells. (TIF) [file ppat.1012727.s001.tif]

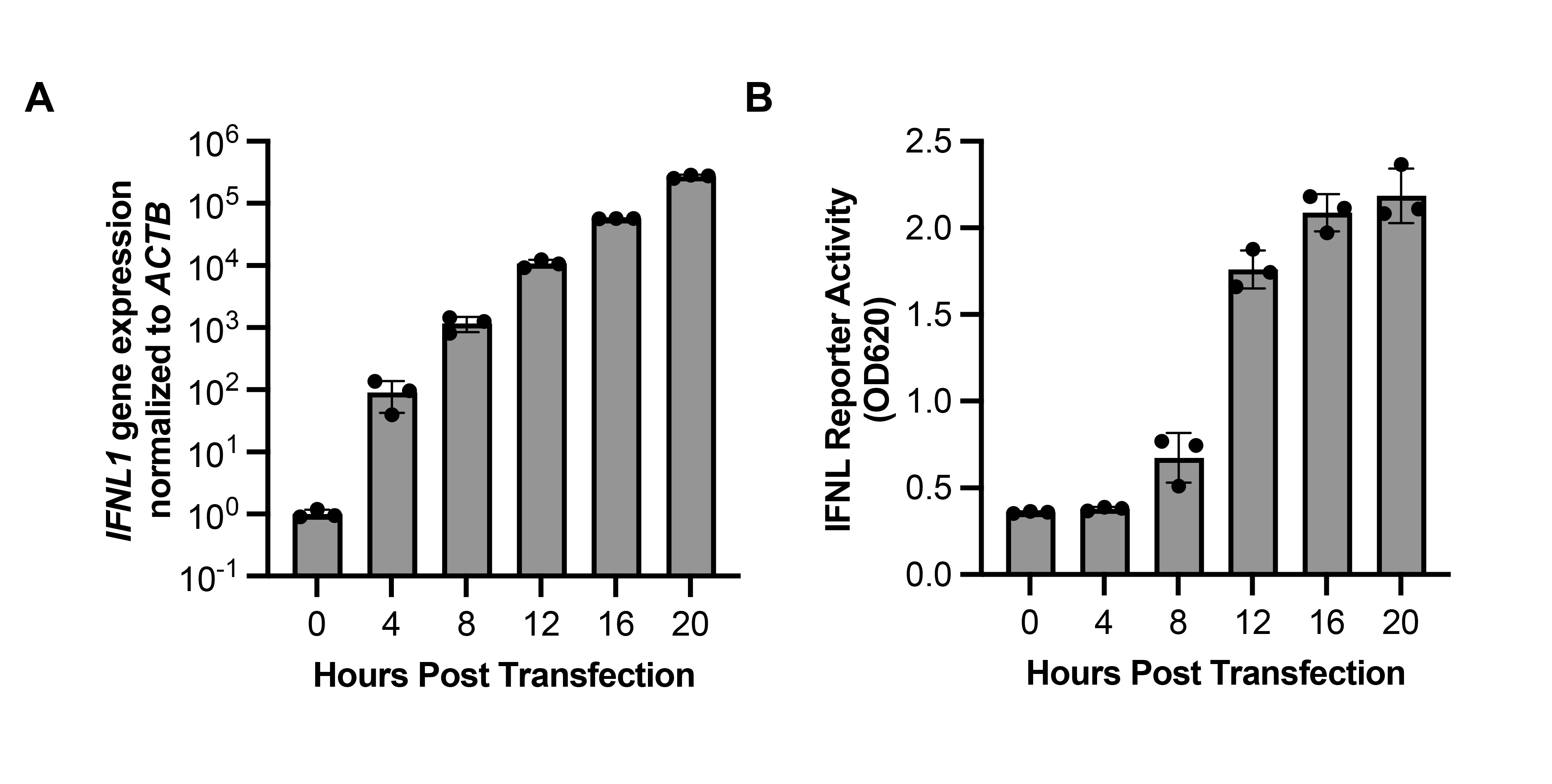

Supplement: S2 Fig — (A) IFNL1 expression from A549 cells transfected with pIC (10 ng/mL) and collected at different timepoints measured by qPCR. (B) Detection of secreted IFNL in supernatant from pIC transfected A549s at different timepoints. Data are shown as mean with SD; N = 3 cell culture wells. (TIF) [file ppat.1012727.s002.tif]

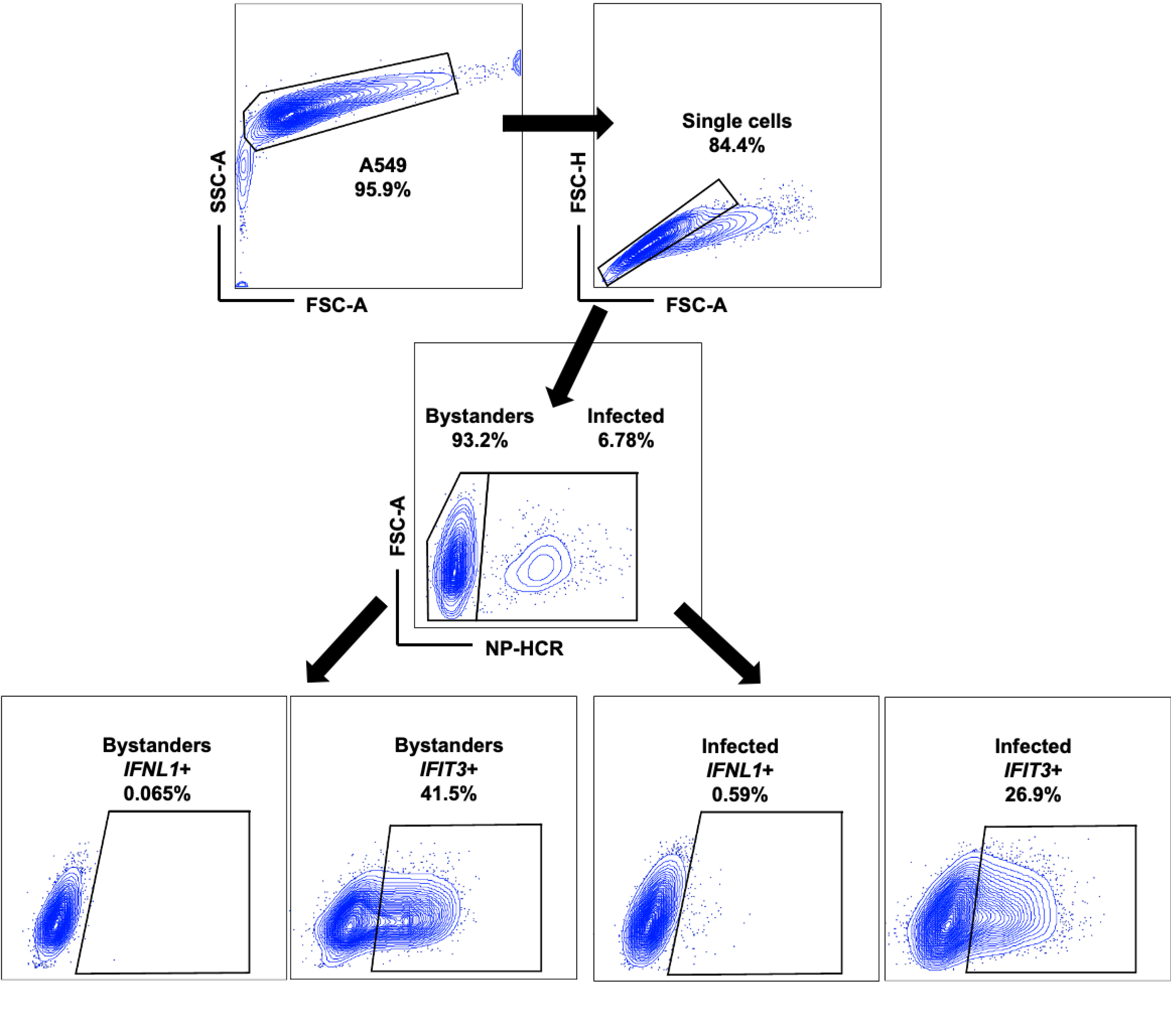

Supplement: S3 Fig — Expression of IFNL1 and IFIT3 mRNA in A549 cells infected with Cal07 at MOI 0.1 NPEU using HCR-flow. (TIF) [file ppat.1012727.s003.tif]

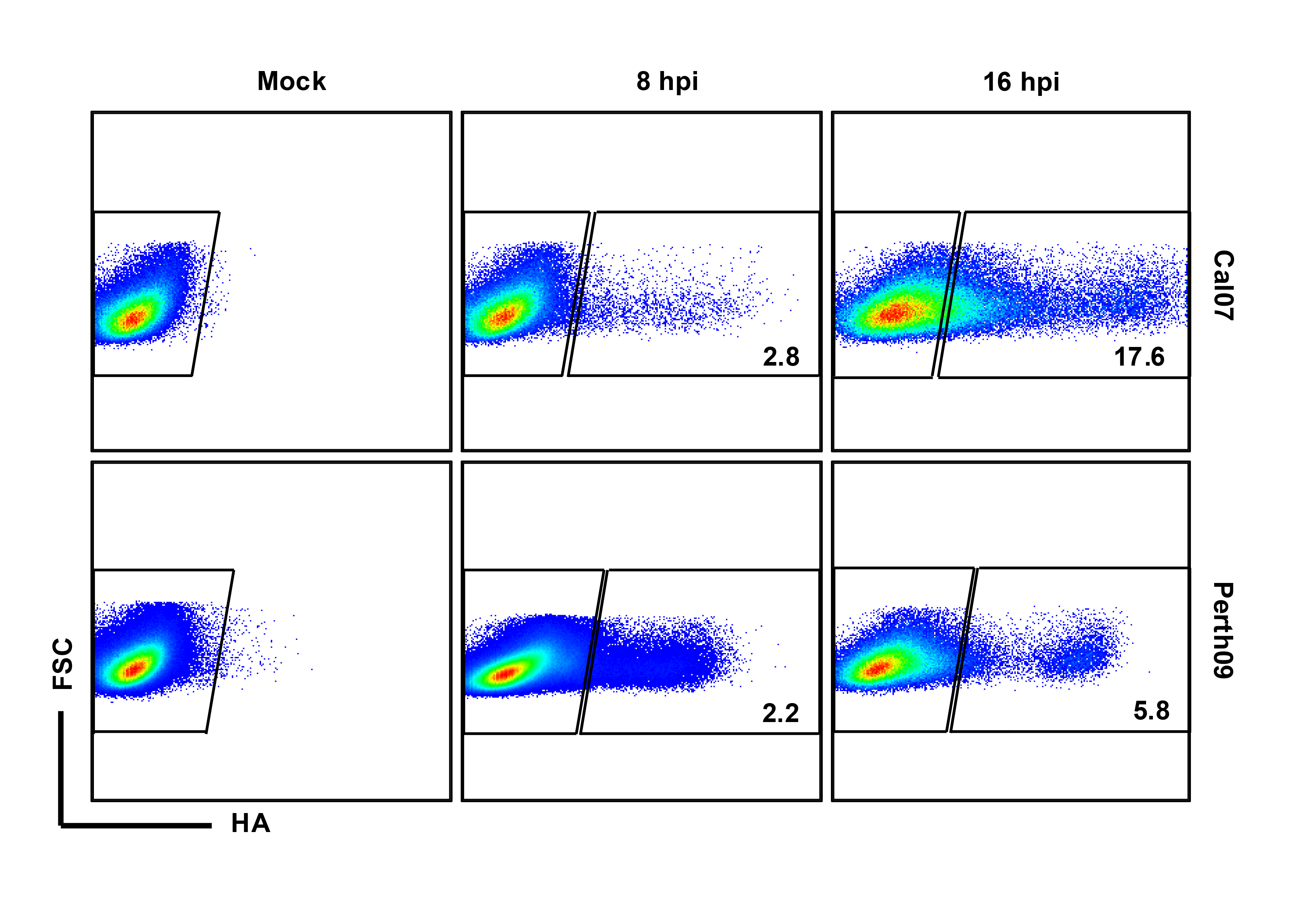

Supplement: S4 Fig — Infected A549s were sorted using FI6v3 (anti-HA) to label infected cells (HA+) or bystander (HA-) prior to preparation of scRNA-seq libraries. (TIF) [file ppat.1012727.s004.tif]

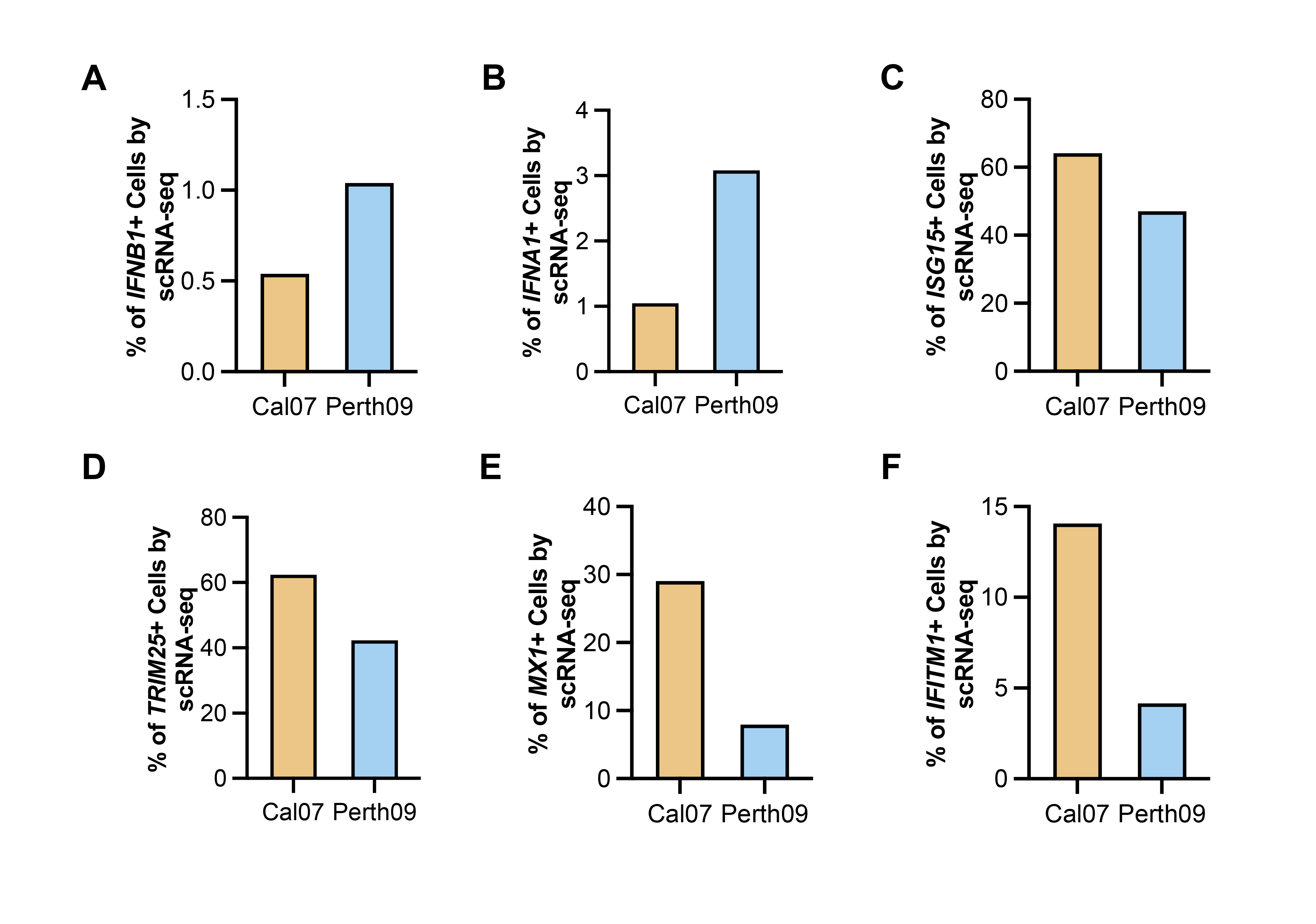

Supplement: S5 Fig — Differences in gene expression between Cal07 or Perth09 infected A549 using scRNA-seq at 16 hpi. Comparison of IFNs (A-B) or ISG (C-F) expression in A549 infected with Cal07 or Perth09. (TIF) [file ppat.1012727.s005.tif]

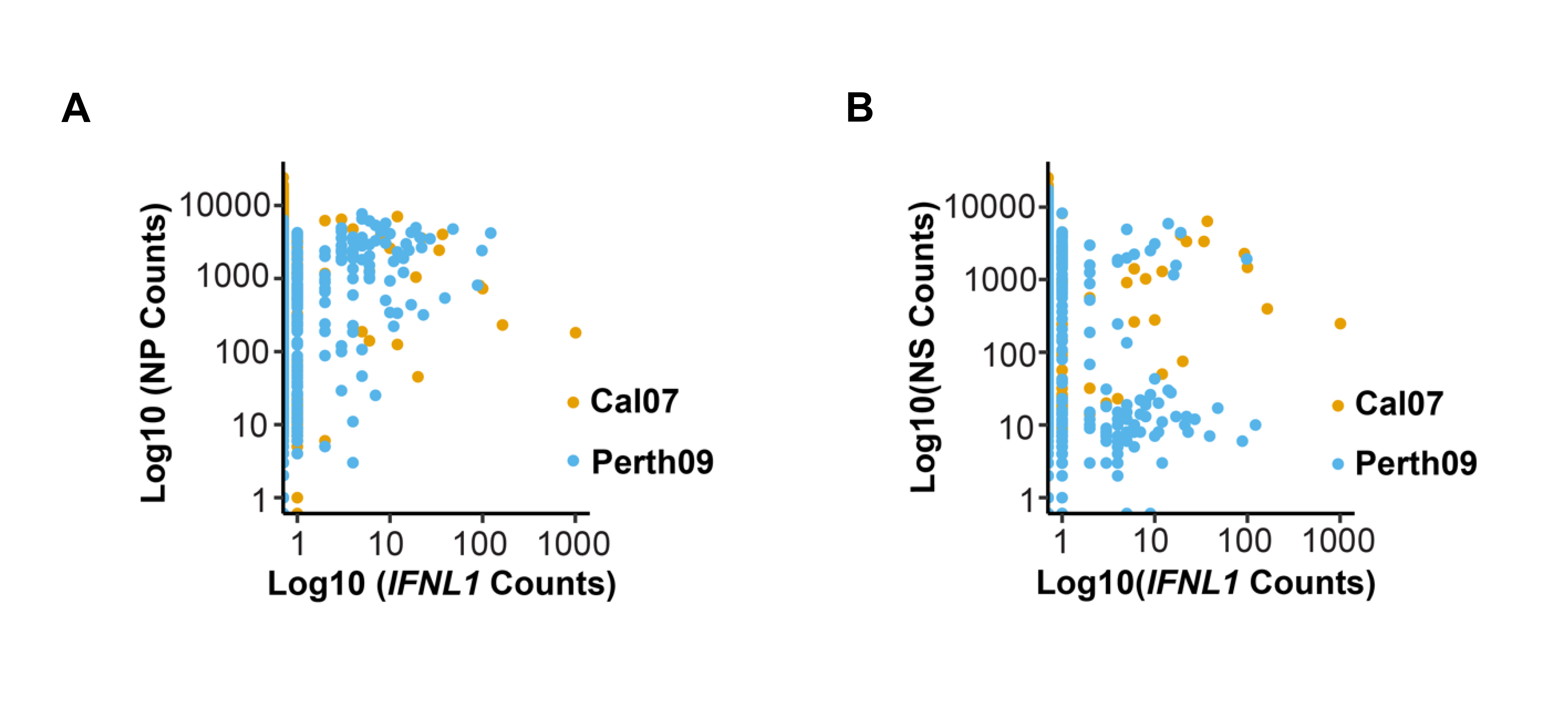

Supplement: S6 Fig — Expression of NP (A) or NS (B) counts compared to IFNL counts across Cal07 and Perth09 libraries. (TIF) [file ppat.1012727.s006.tif]

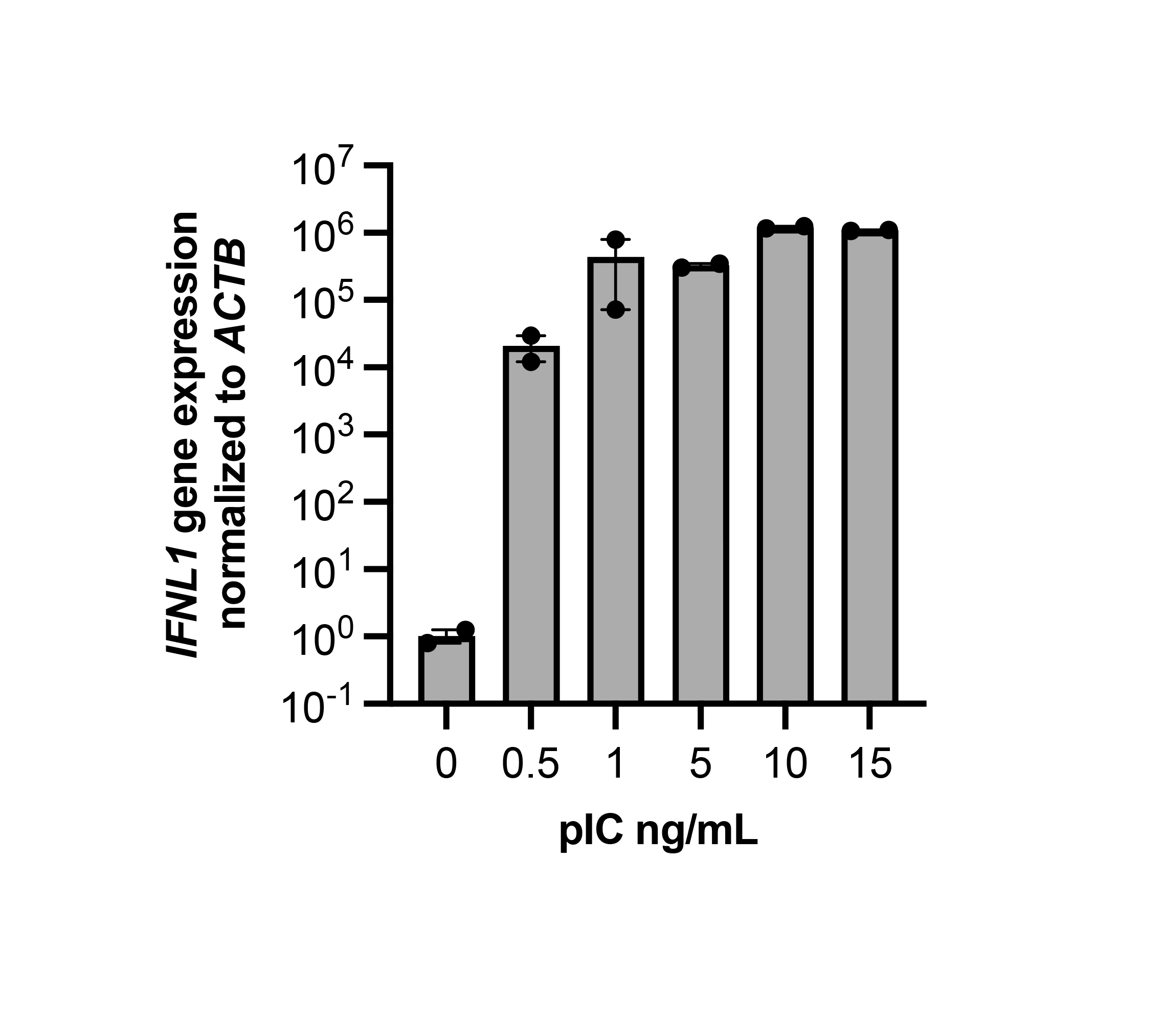

Supplement: S7 Fig — Cells were transfected with pIC to induce immune activation which was quantified by measuring IFNL1 expression. Data are shown as mean with SD; N = 3 cell culture wells. (TIF) [file ppat.1012727.s007.tif]

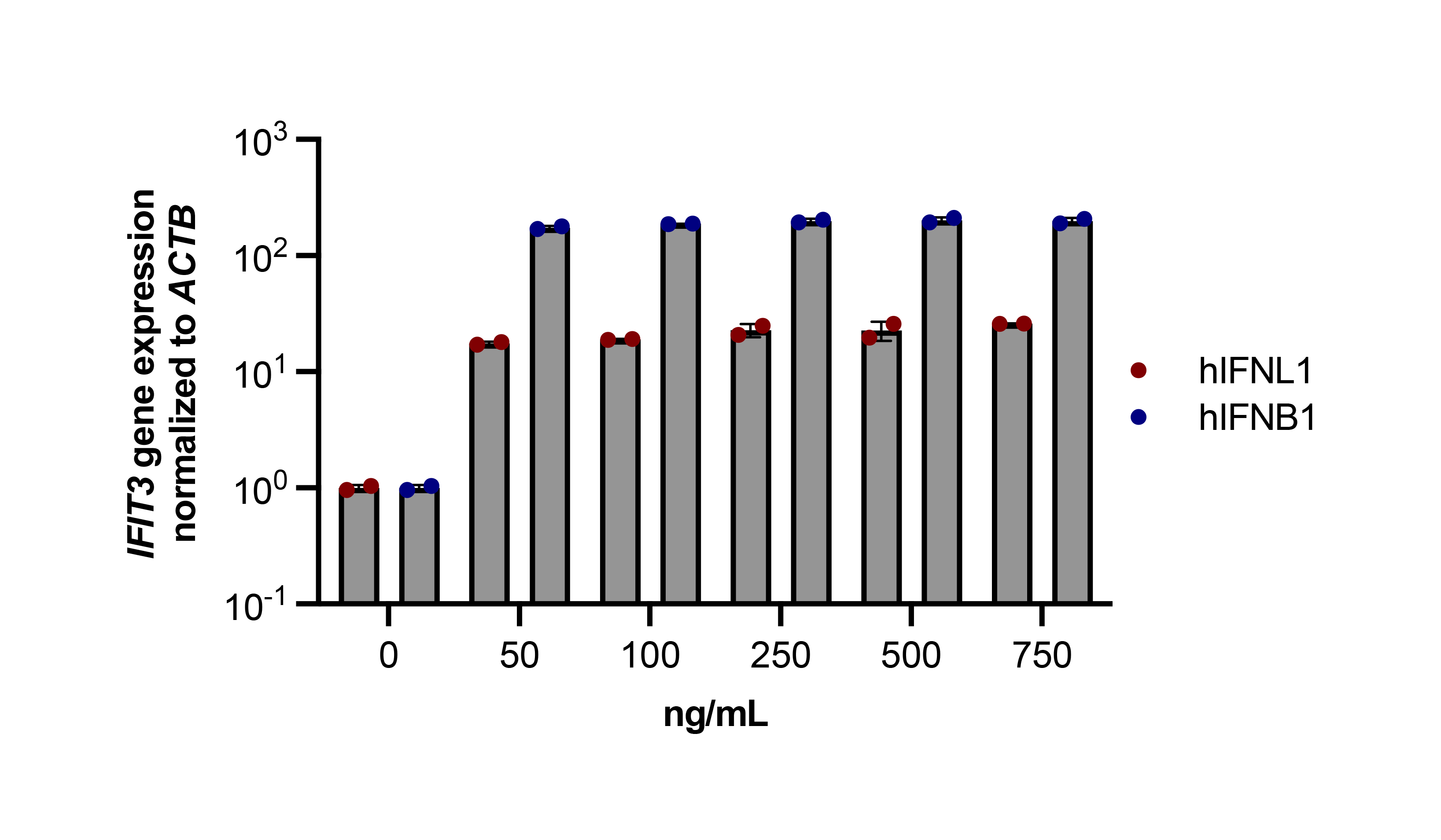

Supplement: S8 Fig — Cells were treated with recombinant IFNs for 16 hrs and ISG induction was measured by quantification of IFIT3 expression. Data are shown as mean with SD; N = 3 cell culture wells. (TIF) [file ppat.1012727.s008.tif]

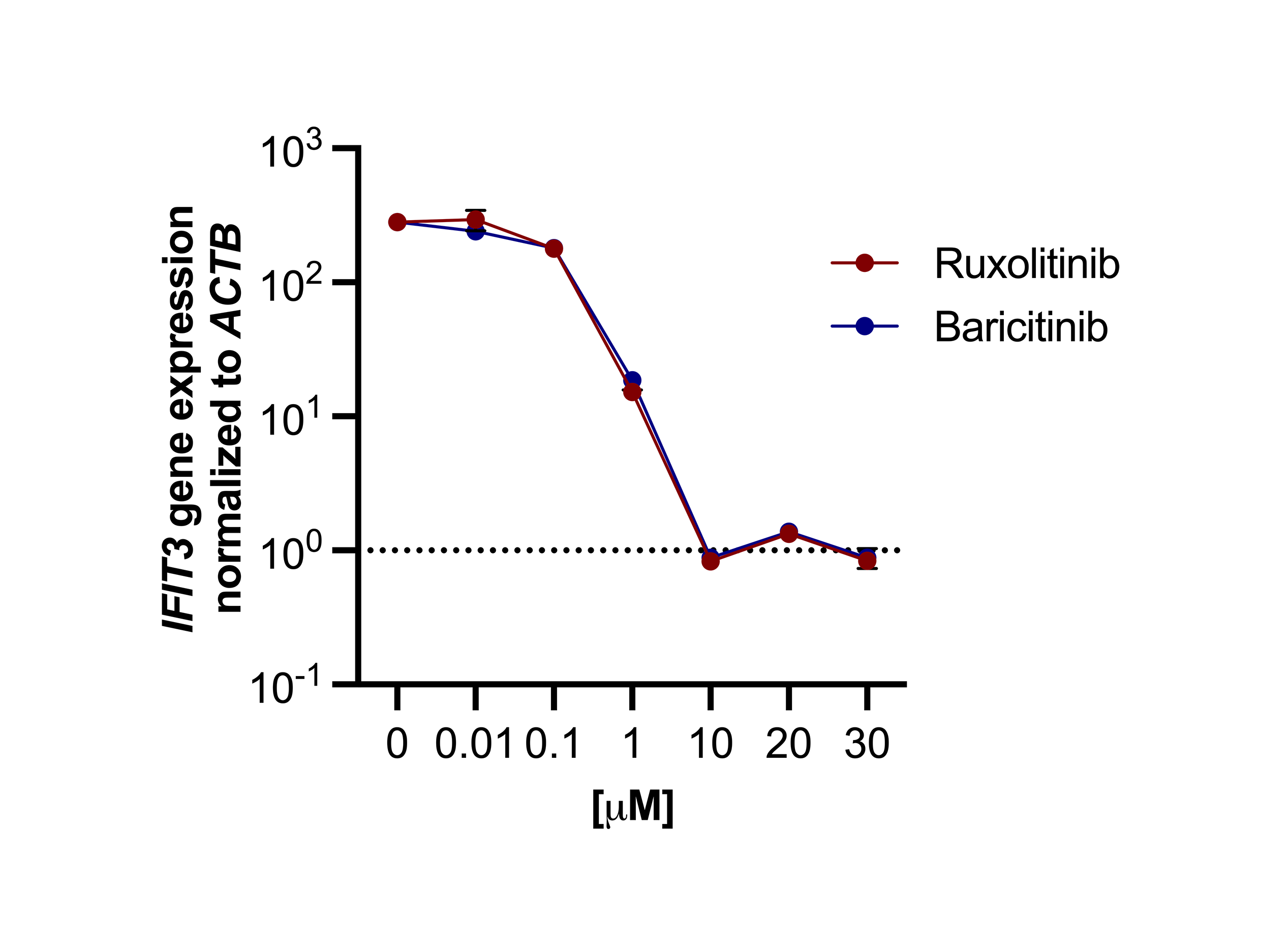

Supplement: S9 Fig — Inhibition was tested by treating A549 for 4 hrs prior immune activation with hIFNB1 (100 ng/ml) and measured by quantifying IFIT3 expression. Data are shown as mean with SD; N = 3 cell culture wells. (TIF) [file ppat.1012727.s009.tif]

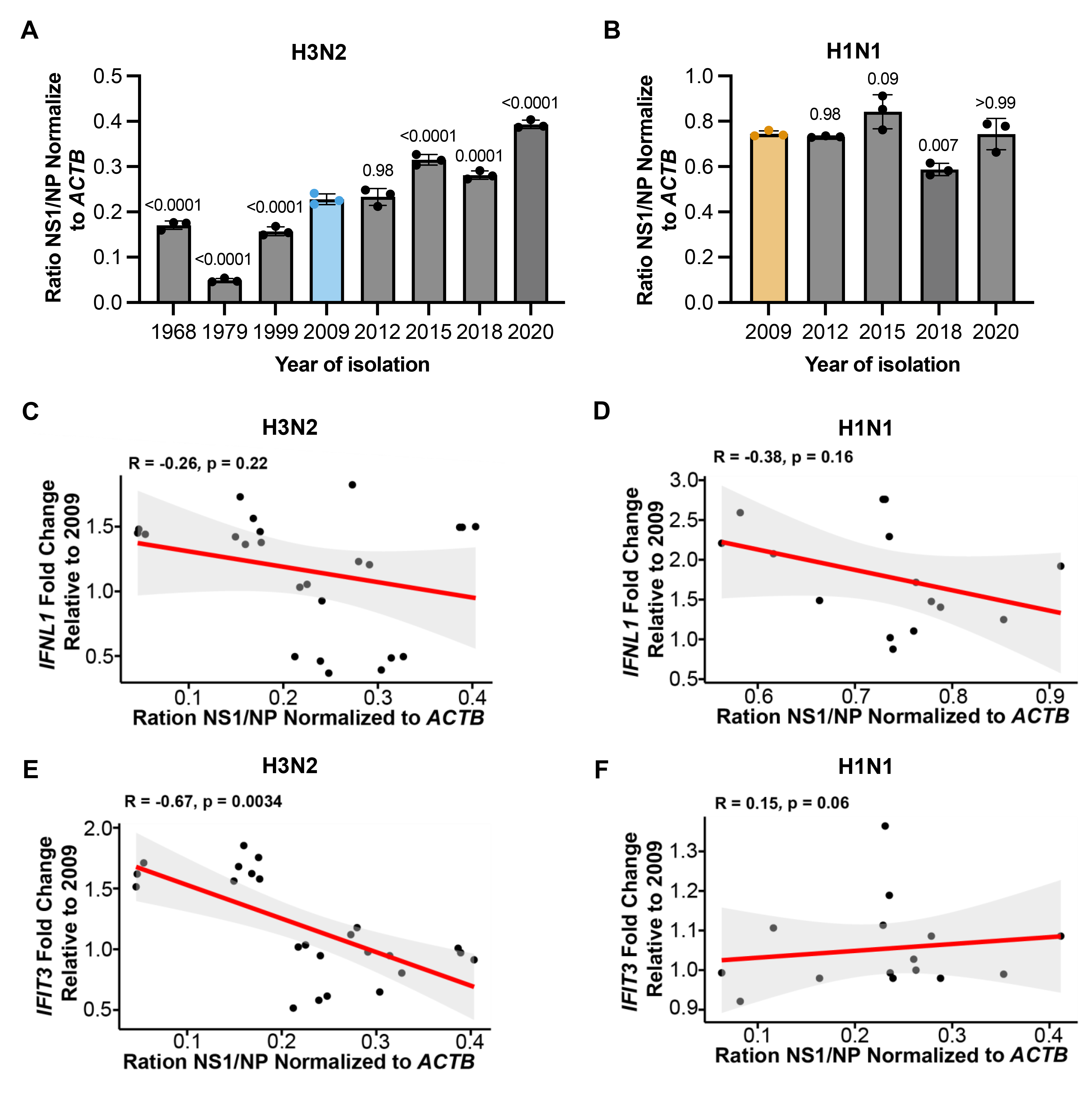

Supplement: S10 Fig — (A) Ratio of NS1 to NP transcripts in A549 cells infected with H3N2 expressing NS from 1968–2020 or (B) H1N1 expressing NS from 2009–2020 at MOI of 0.1 based on NPEU and measured by qPCR. (C) Correlation between IFNL1 fold change and ratio of NS1/NP transcripts in A549 infected with H3N2 expressing NS from 1968–2020 or (D) H1N1 expressing NS from 2009–2020 at MOI 0.1 based on NPEU.(E) Correlation between IFIT3 fold change and ratio of NS1/NP transcripts in A549 cells infected with H3N2 expressing NS from 1968–2020 or (D) H1N1 expressing NS from 2009–2020 at MOI 0.1 based on NPEU. Data are shown as mean with SD with p values indicated on top for comparison to 2009; N = 3 cell culture wells. One-way ANOVA (Dunnett’s Multiple Comparisons test) was used for statistical analysis to compare conditions to 2009 and correlation coefficient and p value was determined by calculating Pearson correlation. (TIF) [file ppat.1012727.s010.tif]
