# Supplementary material for: Seasonal influenza a virus lineages exhibit divergent abilities to antagonize interferon induction and signaling
Source: PLoS Pathog. 2024 Dec 12;20(12):e1012727. doi: 10.1371/journal.ppat.1012727 (PMC11637315; doi:10.1371/journal.ppat.1012727)
Supplement: Attachment — Submitted filename: Joel_PLOSP_reviewers_comments_102224.docx [file ppat.1012727.s011.docx]

**Part II – Major Issues: Key Experiments Required for Acceptance**

Please use this section to detail the key new experiments or modifications of existing experiments that should be absolutely required to validate study conclusions.

**Reviewer #1:** None noted

**Reviewer #2**: Are there kinetic differences in the induction of ISG and IFN between Cal07 and Perth09? While a timecourse is shown for the induction of responses to polyI:C, this is not investigated for either infection. Based on Figure S4, there is more HA present in Cal07 at 8 and 16hrs, and potentially earlier replication, which could impact induction and antagonism of the ISG responses. In Figure 2, it appears as though there may be slightly more NP-HCR following Perth09 at the timepoint analyzed. Whether differential changes in infection kinetics independent of the NS1 segment could partially contribute to changes in responses is not addressed or discussed.

Thank you for the suggestion, we have performed a timecourse experiment with Cal07 or Perth09 infected A549s. The results of this experiment are presented in Figure 3. We observed faster induction of IFN/ISG in Perth09-infected cells compared to Cal07-infected however, qPCR for NP/NS1 show no significant difference in expression at different times with exception of NP at 8 hpi and NS1 at 16 hpi. These experiments suggest that infection kinetics are not playing a major role in the observed IFN/ISG induction profiles for H3N2 and H1N1.

**Part III – Minor Issues: Editorial and Data Presentation Modifications**

Please use this section for editorial suggestions as well as relatively minor modifications of existing data that would enhance clarity.

**Reviewer #1:** For Fig 2 it may be helpful to show the NP and IFNL on the same flow plot to visualize potential correlations between virus levels and IFNL across individual cells. Addition of NS1 would further strengthen this analysis but may be beyond the scope of this manuscript.

Thank you for the suggestion. The short length of NS1 makes it difficult to examine by flow-HCR because we can only target it with a small number of probes. As an alternative, we used our scRNA-seq data to better illustrate the comparison between NP/NS1/IFN which can be found in Figure S6. The data shows a correlation between NP and IFNL1 counts for both viruses however, when comparing NS segment, we can see that IFNL1 positive cells are associated with lower NS counts in Perth09-infected cells but not in Cal07-infected.

Fig. S9 is pretty important to evaluate Fig. 6. Can this be moved into the main figure?

Thank you for the suggestion, we have moved it to the main figure (Fig. 7A,B).

Line 43 - potential typo, assuming it should be NS not NA

Thanks, we have fixed this error.

Line 103 - Several other groups have also demonstrated heterogeneity of virus-induced IFN induction and should be cited (PMIDs 30626670, 33507952, 33730024).

Thank you for the suggestion, we have added them to the text line 102-103.

Line 111 - the authors discuss the high cost of single cell, is it worth also noting the low sensitivity and stochasticity? Which may be overcome by their approach?

Thank you for the comment, we have added more information about the limitations associated with scRNA-seq in line 111-112.

Number of samples/replicates is missing for some data

Thank you for the feedback, we have specified the number of replicates in figure legends.

**Reviewer #2:** How many times each experiment was performed and individual replicates (or error bars) are not included for several figure panels throughout and should be added.

Statistics and data plotted are based on culture wells analyzed. Were experiments repeated multiple times? If so, showing replicates (even in a supplemental figure) would be helpful to assess heterogeneity of the system/assay between infection experiments. At a minimum, potential heterogeneity across infection experiments could be mentioned in the text with data not shown.

Thank you for the suggestions, we have modified the figures to show mean with standard deviation as error bars. We have added more information about the number of replicates and independent experiments to figure legends. Additionally, we have described the number of replicates and independent experiments and addresses the challenges with heterogeneity in immune induction when comparing A549s at different passage numbers in lines 167-170.

It is unclear why statistics are included on some graphs where results are described as different, but not others. Statistical analysis should be updated throughout. Additionally, post-hoc analysis for data in Figure 6 to know which viruses/years have statistically significant changes in IFIT3 relative induction compared to the 2009 isolates.

Thank you for the feedback, we have added statistics to the comparisons performed across the study with exception of Figure 2E were we calculated the percentage for the entire scRNA-seq library for Perth09 or Cal07 resulting in one single value per comparison. For Figure 7 we have performed One-way ANOVA (Dunnett’s Multiple Comparisons test*)* to compare all isolates to 2009. We have presented the p values obtained from the comparison on top of each column in the graph.

In Figure 5F, it appears as though Ruxolitinib significantly reduces IFITM3 expression in both viral infections despite more + signal remaining in Perth09-infected wells. This suggests ISG antagonism is at least partially dependent upon JAK/STAT signaling for H3N2. Language should be softened in the manuscript to address this.

Thank you for the feedback, we have addressed this in the text by changing the Figure 6 legend.
